# Medical Teaching

**Published:** 1866-07

**Authors:** 


### Selections.

---

### MEDICAL TEACHING.

The following from the editorial columns of the *Boston Medical and Surgical Journal*, upon the subject of medical teaching and study, we regard as so directly to the point, that we suggest for it a careful perusal. So broad and extensive has become the field of medical science, that no one mind has the power of comprehending the whole, much less of elaborating it. The present rapid development in medical science, is largely, if not chiefly, owing to the fact that there is a focalizing of attention and study, upon particulars or specialities. Men do not now, as formerly, rest satisfied with the attainment of general principles, but taking these as a starting point, strike out in a definite channel, and in this way, make the wonderful discoveries and developments, which, during the past few years, have followed each other in such rapid succession.

Formerly, those who were regarded as great men were rare, for it required giant intellects to grapple with the whole subject, embrace it, and carry it forward. Now, a far greater proportion may become deservedly great, by selecting a particular department, and concentrating upon it his whole power, and thus bring out its fullest development.—Ed.

